# Exploring work productivity loss in patients with inflammatory bowel disease

**DOI:** 10.2144/fsoa-2022-0034

**Published:** 2023-06-07

**Authors:** Sahar Nasr, Wafa Dahmani, Hanene Jaziri, Aya Hammami, Aida Ben Slama, Wafa Ben Ameur, Nour Elleuch, Mahdi Ksiaa, Ali Jmaa

**Affiliations:** 1 Department of Gastroenterology, Sahloul University Hospital, University of Sousse, Tunisia

**Keywords:** Crohn’s disease, inflammatory bowel disease, low to middle income country, occupational medicine, quality of life, Tunisia, ulcerative colitis, work productivity and activity impairment

## Abstract

**Aim:**

We aimed in this study to evaluate the impact of inflammatory bowel disease (IBD) on patients’ professional life and to determine predictors of severe work productivity loss (WPL).

**Materials & methods:**

A cross sectional study including patients with a confirmed diagnosis of IBD. Work productivity was evaluated with the work productivity and activity impairment score.

**Results:**

Severe absenteeism and WPL were found in respectively 7 (5.1%) and 54 (39.1%) patients. In multivariate analysis, the following features were found to be independently associated with severe WPL: penetrating Crohn’s disease (p: 0.001, OR: 6), anemia (p: 0.031, OR: 3.23), diarrhea (p < 0.001, OR: 11.23) and a secondary level of education (p: 0.003, OR: 1.95).

**Conclusion:**

Our results show that IBD have a substantial effect on patients’ professional life.

## Plain language summary

Inflammatory bowel diseases (IBD) are chronic conditions that cause inflammation in the digestive system. We wanted to know how IBD affects people’s ability to work and what factors contribute to work difficulties. We asked adults with IBD about their education, work conditions and medical history. We found that a significant number of patients experience severe work productivity loss and absenteeism. Factors such as certain types of IBD, anemia, diarrhea and lower education level were linked to more severe work problems. These findings emphasize the impact of IBD on work life and highlight the importance of addressing these challenges in patient care.

Crohn’s disease (CD) and ulcerative colitis (UC), known as inflammatory bowel diseases (IBD), are chronic inflammatory diseases of the GI tract usually diagnosed in early adulthood, a critical age in which individuals usually start their professional life. IBDs are characterized by their chronicity and their relapsing and remitting course with unpredictable flares, debilitating symptoms and frequent complications [1,2]. This results in high morbidity and healthcare costs [3,4]. In fact, loss of work productivity is frequently reported in patients with IBD [5–7]. In addition, patients with IBD are less likely to be hired and are thought to remain unemployed for longer periods of time when compared with the general population [8,9]. In addition, IBD complications such as surgery and frequent hospital admissions can lead to layoff and difficulties in keeping jobs and in career advancement. Moreover, the performance of patients with IBD seems to be substantially compromised even when they attend their work stations. In fact nearly thirty percent of patients with IBD report a lack of motivation at work, social isolation and concentration difficulties [10]. The results of the few published studies analyzing predictive factors of work productivity loss (WPL) and work disability are controversial and they include early onset of IBD, female gender, active perineal disease and fatigue [11–15]. To the best of our knowledge, most of the studies estimating IBD impact on work were conducted in developed countries where work conditions and practices are different from those of low-to-middle income countries. In fact, the longer work hours, the poor work conditions, especially for patients working in factories, as well as poor wages, work environment and sanitation might account themselves for severe absenteeism. Therefore, we aimed in this study to determine the impact of IBD on Tunisian patients’ professional life and to identify the predictive factors of severe WPL in these patients.

## Materials & methods

This is a cross sectional study conducted during a 3 month period including patients with a confirmed diagnosis of IBD admitted or presenting for a routine visit to the Gastroenterology Department of Sahloul University Hospital, a tertiary referral center in Tunisia. The diagnosis of IBD was based on clinical, endoscopic, radiological and histopathological features. We have included consenting patients aged 18 years or higher, who have been working for at least 1 year or had a professional activity. The sample size was estimated with the percentage of absenteeism reported in Sara Van Gennep *et al.*’s study [11]. The minimum sample size was 162 at 95% power and α ¼ 0.01 error levels. We excluded patients with unclassified IBD, patients taking experimental treatments and patients with non-IBD related complete work disability with the latter defined by having a physical or mental condition that precludes substantial gainful activity.

### Instruments

To evaluate the impact of the IBD on patients’ professional life, we have collected data concerning patients’ careers, features of their current jobs and difficulties encountered in their daily professional activities. In addition, we used the «Work Productivity and Activity Impairment» (WPAI) score to evaluate work productivity. The validity, reliability and responsiveness of the WPAI have been proven in both CD and UC patients [16,17]. The score contains six questions evaluating the following features during the 7 days preceding interrogation: Question (Q)1 employment status; Q2 hours missed due to IBD; Q3 hours missed due to other reasons; Q4 insured hours of work during the last week; Q5 the degree to which IBD is affecting work productivity on a scale from 0 to 10 (with ten referring to maximum impairment); and Q6, the degree to which IBD is affecting non work related daily life activities (from 0 to 10). The answer of questions from Q2 to Q6 absenteeism, presenteeism and WPL were calculated based on the following formula respectively: Q2 / (Q2 + Q4); Q5 / 10; Q2 / (Q2 + Q4) + [(1 - (Q2 / (Q2 + Q4))) × (Q5 / 10)]. The scores were expressed as percentages with higher values indicating more severe work impairment.

### Data collection

The questionnaires were answered either par the patients themselves or during meetings with doctors. In cases where patients reported IBD-related absenteeism, they were asked to mention the reasons. IBD-related data were collected from patients’ medical records and endoscopy reports.

### Outcomes & definitions

The primary outcome of the study was to identify the rate of work absenteeism, presenteeism and WPL. The secondary outcome was to determine predictors of severe WPL. Absenteeism is defined by the time missed due to IBD with severe absenteeism defined with a percentage of missed hours due to IBD of 50% or higher. Presenteeism is defined by a loss of efficacy due to IBD in spite of physical presence at work. Severe presenteeism was defined by ≥50% productivity loss at work. WPL refers to the combination of absenteeism and presenteeism. It is a measure of the extent to which a health problem affects an employee’s ability to perform their job duties effectively, including both the amount of time missed from work and the reduced productivity while at work. Severe WPL was defined with a percentage of 50% or higher. In fact, this threshold of productivity loss is often associated with a greater impact on the quality and quantity of work completed, as well as increased healthcare costs and disability and was approved by a panel of experts consulted during the development of the WPAI questionnaire [18].

### Ethical consideration

The principles of the Declaration of Helsinki were respected along of this study. Informed written consent was obtained from all patients included in the study.

### Statistical analysis

Continuous variables were expressed as mean ± standard deviation (SD) for those following a normal distribution and as median and interquartile range (IQR) for those that failed the normality test. Qualitative variables were expressed as number and percentages and compared by the Chi-squared test or Fisher’s exact test. Inter-group comparisons of continuous variables were analyzed by means of a student *t*-test and the Mann–Whitney test for variables with non-normal distribution failed the normality test. A multiple logistic regression analysis was carried out to identify predictors of outcomes; unemployed patients were excluded from this part of the analysis. During the development of the final model, we investigated all possible combinations of candidate variables whose p-values were <0.2 in univariate analysis. A p-value < 0.05 was considered statistically significant. The statistical tests applied were two-tailed. Analysis was performed using the statistical software program SPSS for Windows version 22.

## Results

**Figure 1.**
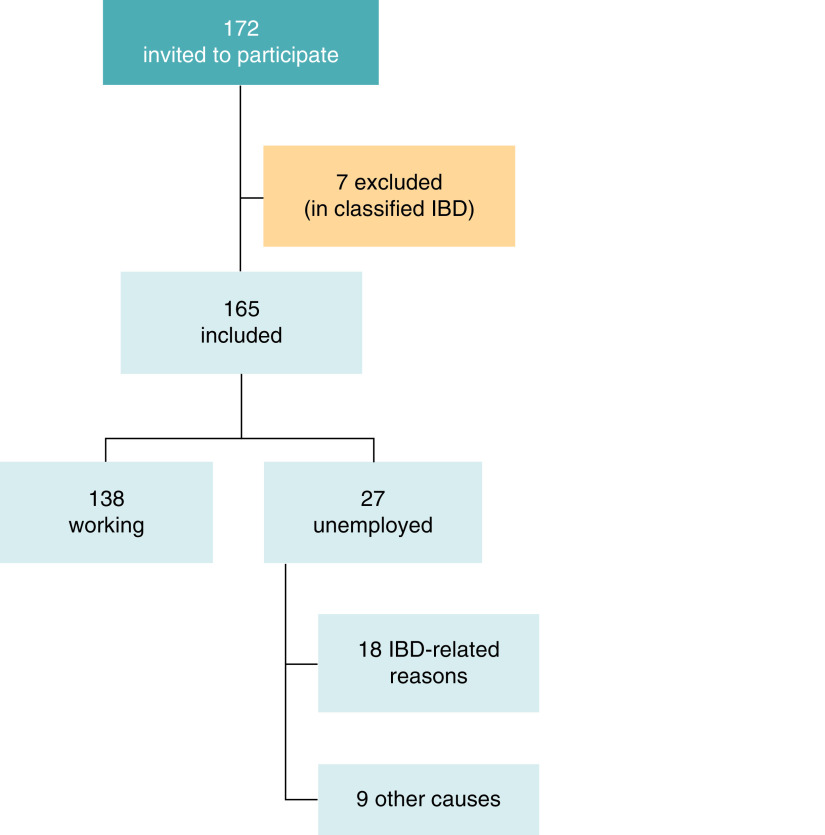
Study flowchart. IBD: Inflammatory bowel disease.

The study flowchart is depicted in Figure 1. All patients approached consented to participate.

During the study period, we have included 165 patients with a median age of 34 years (IQR) [27–42]. The sex ratio (M/F) was 1.1. Overall, 73.9% of patients had CD and 26.1% had UC. The most common disease location for patients with CD was ileocecal (n = 59, 48.4%).

The most common disease behavior was non penetrating and non structuring (n = 59, 48.4%) followed by penetrating CD (n = 53, 43.4%).

At the time of interrogation, 26 (15.8%) patients were taking mesalazine as monotherapy, 74 (44.8%) were taking azathioprine as monotherapy while 57 (34.5%) patients were using azathioprine in combination with anti-TNF. Among these patients, three were using adalimumab and 54 were using infliximab. Only eight patients (4.8%) were not receiving maintenance therapy. At the time of interrogation, 138 were professionally active. Among the rest, 66.7% (n = 18) had stopped working due to IBD-related problems. The demographical and IBD features and those related to patients’ professional life are summarized in (Table 1 & 2) respectively.

**Table 1.** Patients' demographical and inflammatory bowel disease-related features.

Features		
Age median, (IQR)	34	(27–42)
Male, n, %	8	52.7%
Education levels, n, % Elementary Secondary Postsecondary	21 103 40	12.7% 62.4% 24.2%
Duration of IBD median, (IQR)	4	(2–6)
Follow-up duration ≥5 years, n, %	56	33.9%
Crohn disease, n, %	122	73.9%
Location of Crohn's disease, n, % Ileum-colon Terminal ileum Colon Upper gastro-intestinal tract Anoperineal lesions	59/122 54/122 10/122 12 33	48.4% 44.3% 8.2% 9.8% 27%
Type of Crohn disease, n, % Inflammatory Penetrating Structuring	59 53 31	48.8% 43.4% 25.4%
Ulcerative colitis, n, %	43	26.1%
Location of ulcerative colitis, n, % Proctitis Left colon Pancolitis	7/43 4/43 32/43	16.3% 9.3% 74.4%
Resection performed, n, % Colectomy Colic resection Ileocecal resection	51 11 4 36	30.9% 6.7% 2.4% 21.8%
Stoma, n, %	4	2.4%
Associated primary sclerosing cholangitis, n, %	4	2.4%
Active arthralgia, n, %	33	20%
Anemia, n, %	82	49.7%
Clinical and endoscopic remission, n, %	42	25.5%
Admitted at the time of interrogation, n, %	38	23.5%
Main hospital admission causes, n, % Acute severe colitis Bowel obstruction Intra-abdominal abscess Severe disease flare	10/38 4/38 11/38 5/38	26.3% 10.5% 28.9% 13.2%
Treatment adjustment during the last three years, n, %	37	26.8%
Current treatments, n, % Mesalazine as monotherapy Azathioprine as monotherapy Azathioprine + anti-TNF	26 74 57	15.8% 44.8% 34.5%
Steroid-dependence, n, % Steroid resistance, n, %	37/115 5/115	30% 4.3%

IBD: Inflammatory bowel disease; IQR: Interquartile range.

**Table 2.** Patients’ professional life features.

Features		
Current status, n, % Active Retired Unemployed	138 0 27	83.6% 0 16.4%
Position, n, % Blue collar work Administrative Teaching Other	67/138 29/138 9/138 33/138	48.5% 21% 6.5% 23.9%
Bread winner position, n, %	62	44.9%
Self-employed, n, %	31	22.5%
Private sector, n, %	96	69.6%
Working hours per week, n, % <40 ≥40	36 102	26.1% 73.9%
Frequent traveling, n, %	16	11.6%
Constant working by day, n, %	131	94.9%
Constant working by night, n, %	4	2.9%
Night shifts, n, %	3	2.1%
Impact on choice of career, n, % Not concerned Yes (took IBD into consideration when choosing career) No	92 7/46 39/46	66.7% 15.2% 84.8%
Impact on career, n, % Relocation to a different work post Changed career	11 16	7.9% 11.6%

IBD: Inflammatory bowel disease.

### WPL & daily life impairment

**Figure 2.**
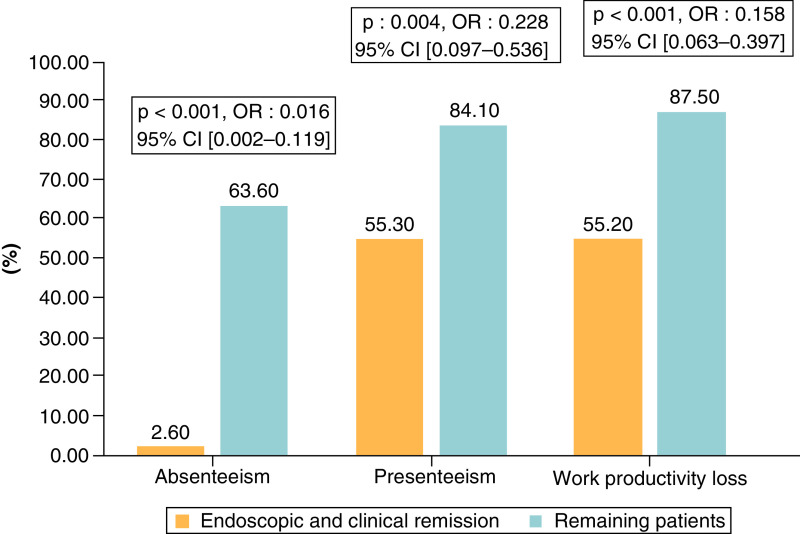
Prevalence of absenteeism, presenteeism and work productivity loss in patients with and without clinical and endoscopic remission. CI: Confidence interval; OR: Odds ratio.

Sixty-six patients (47.8%) had to miss work in the past week due to IBD-related reasons. Among these patients, the median absenteeism rate was 12.9%, IQR: (9–22.5%). Presenteeism was reported by 98 (71%) patients, the median presenteeism rate was 50%, IQR: (30–70%). Overall, WPL was reported in 107 patients (77.5%) with a median WPL of 48.6%, IQR: (25.3–74.3%). Severe absenteeism and WPL were reported by respectively 7 (5.1%) and 54 (39.1%) patients. Overall, 108 (65.4%) patients (both employed and unemployed) reported daily activities impairment secondary to IBD with an impact estimated to be 50% or higher in 27.9% of cases (n = 54). When comparing WPAI features between CD and UC patients, we found a significant difference in severe absenteeism rates and severe IBD impact on daily activities rates between the two groups (Table 3 shows WPAI score results). As expected, the prevalence of patients with absenteeism, presenteeism and WPL were significantly lower in patients with endoscopic and clinical remission when compared with the remaining patients (p: < 0.001, 0.004, <0.001 respectively) (Figure 2).

**Table 3.** Results of the work productivity and activity impairment questionnaire in our population.

Features	Population			p-value
	Total	Crohn’s disease	Ulcerative colitis	
Missed work due to IBD-related reasons, n, %	66, 47.8%	45, 45%	21, 55.3%	0.281
Missed work for other reasons, n, %	24, 17.4%	19, 19%	5, 13.2%	0.228
Absenteeism Median/IQR	12.9%, (9%–22.5%)	14.3% (10%–22.8%)	10.9% (6.7%–35.5%)	0.620
Severe absenteeism, n, %	7, 5.1%	2, 2%	5, 13.1%	0.017; OR: 7.42, CI 95% (1.375–40.099)
Reported presenteism, n, %	98, 71%	72, 72%	26, 68.4%	0.679
Presenteeism rate Median, IQR Or Mean, SD	50%, (30%–70%)	50%, (30%–70%)	55%, 27.2%	0.349
Severe presenteeism, n, %	54, 39.1%	37, 37%	17, 44.7%	0.789
Presented work productivity loss, n, %	107, 77.5%	78, 78%	29, 76.3%	0.832
Work productivity loss rate Median/IQR	48.6%, (25.3%–74.3%)	48% (25.2%–72.9%)	52.4% (25.3%–81.8%)	0.377
Severe work productivity loss, n, %	54, 39.1%	39, 39%	15, 39.5%	0.959
Reported IBD impact on daily life activities, n, %	108/165, 65.4%	79, 64.8%	29, 67.4%	0.750
Reported severe IBD impact on daily activities, n, %	54/165, 27.9%	34, 27.9%	20, 46.5%	0.025, OR: 2.25, 95CI (1.098–4.615)

CI: Confidence interval; IBD: Inflammatory bowel disease; IQR: Interquartile range; OR: Odds ratio; SD: Standard deviation.

### Patients reported causes of absenteeism

**Figure 3.**
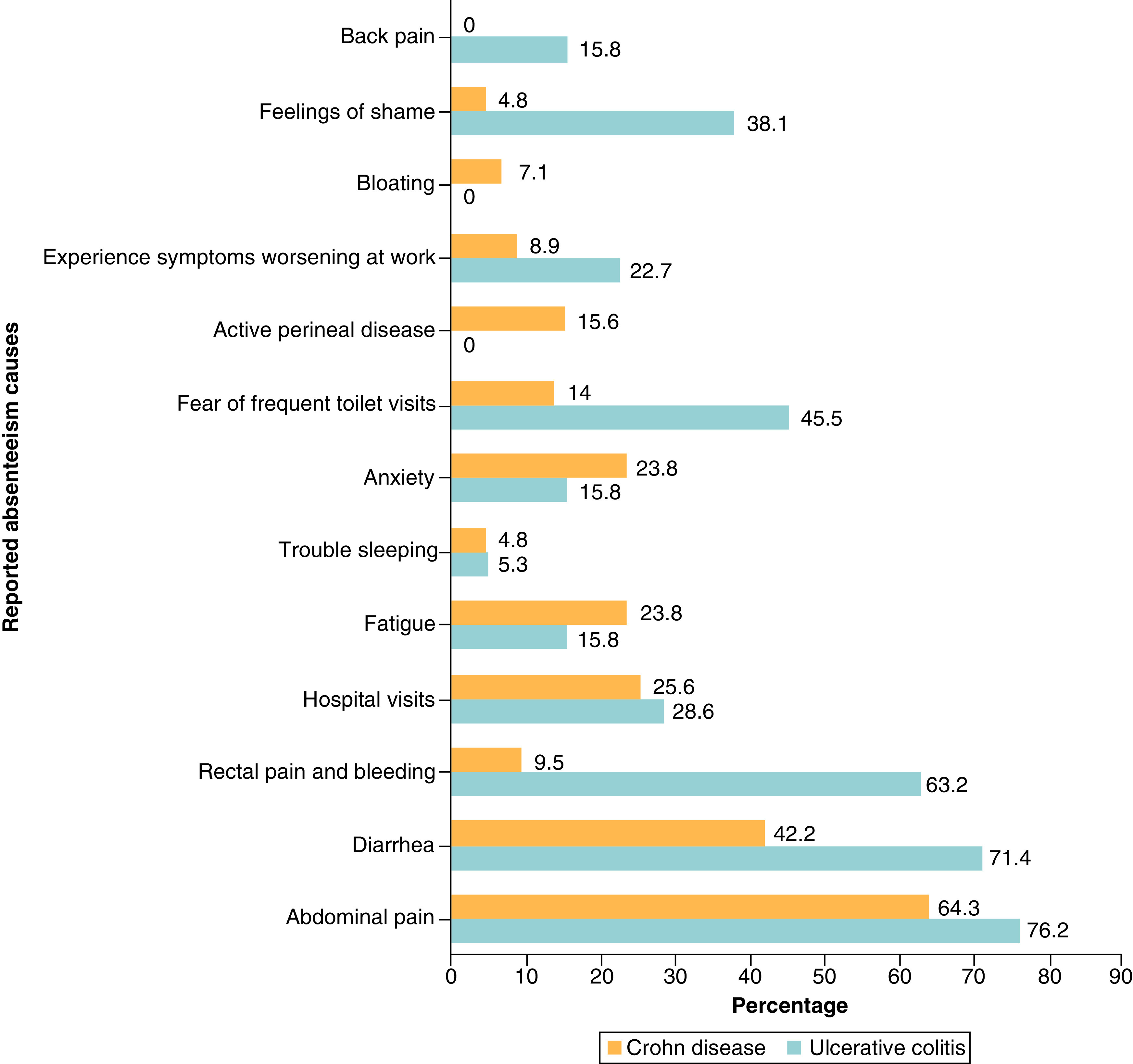
Reported absenteeism causes.

The most common causes of absenteeism were abdominal pain (n = 43, 65.2%) followed by diarrhea (n = 34, 50.7%), rectal pain and bleeding (n = 17, 25.4%) and hospital visit (n = 17, 25.4%). Other causes included feeling ashamed of the symptoms (n = 12, 17.9%), fatigue (n = 14, 20.9%) and trouble sleeping (n = 3, 4.5%). In all cases, patients reported more than one reason for absenteeism. When we analyzed the reported absenteeism causes in patients with CD and those with UC separately, we have found that the following reasons were more frequently reported by patients with UC with a statistically significant difference: rectal pain and bleeding (63.2 vs 9.5%, p < 0.001), fear of frequent toilet visits (45.5% vs 14%, p:0.003) and feeling ashamed (38.1 vs 4.8%, p: < 0.001) (Figure 3).

### Predictive factors of severe work productivity loss

In multivariate study, penetrating CD was identified as an independent predictor of severe WPL (OR: 6.00, 95% CI: 2.120–16.996) (Table 4). On the other hand, the disease location (for both CD and UC), treatments received and history of surgery did not affect this outcome. Although patients with endoscopic and clinical remission had lower severe WPL in univariate analysis (<0.001), this feature did not reach statistical significance in multivariate analysis. With regard to hemoglobin level, the presence of anemia was predictive of severe WPL in multivariate analysis (OR: 3.253, 95% CI: 1.116–9.488). We further analyzed the relationship between severe WPL and IBD-related reasons for absenteeism and presenteeism. While symptoms such as subocclusive syndrome, diarrhea, abdominal pain and anxiety were associated with severe WPL in univariate analysis, only diarrhea reached statistical significance in multivariate analysis (p < 0.001; OR: 11.233; 95% CI: 3.630–34.762). When looking at labour features, we found that work sectors, job nature, traveling obligations and working hours ≥40 years did not affect outcomes. However, we have found that a secondary level of education was associated with higher severe WPL rates.

**Table 4.** Factors associated with severe work productivity loss in inflammatory bowel disease patients with professional activities.

Features	Univariate analysis			Multivariate analysis	
	Severe work productivity loss		p-value	Severe work productivity loss	
	Yes	No		p-value	Odds ratio, 95% CI
Male	30	48	0.854		
Crohn disease	39	61	0.959		
Penetrating Crohn disease	26	20	0.001	0.001	6.00 [2.120–16.996]
Structuring	13	14	0.086		
Anoperineal disease	10	15	0.711		
Ileal Crohn disease	11	32	0.036		
Ileo-colic Crohn disease	25	25	0.008		
Follow-up duration ≥5 years	20	36	0.515		
Treatment switch during the last three years	14	23	0.292		
Underwent surgery	23	25	0.122		
Stoma	3	1	0.524		
Azathioprine	20	42	0.135		
Steroid dependence	6	24	0.002		
Steroid resistance	0	2	0.501		
Combotherapy	25	23	0.018		
Infliximab	25	21	0.01		
Active perinanal disease	4	3	0.432		
Anemia	44	44	0.001	0.031	3.253 [1.116–9.488]
Endoscopic and clinical remission	4	34	<0.001		
Hospital admission	31	1	0.001		
Symptoms Diarrhea Abdominal pain Subocclusive syndrome Rectal syndrome Fatigue Trouble sleeping Anxiety Bloating	28 34 7 11 6 2 3 0	13 24 31 6 8 1 11 3	<0.001 <0.001 0.002 0.398 0.373 0.1 0.004 0.081	<0.001	11.233 [3.630–34.762]
Education level: secondary	40	44	0.011	0.003	5.195 [1.763–15.308]
Blue collar work	33	34	0.175		
Private sector	37	59	0.830		
Frequent traveling	11	5	0.01		
Working hours per week ≥40	34	68	0.019		

## Discussion

This study has highlighted the high burden of IBD in the Tunisian population. Overall, WPL was reported by 77.5% of our patients. The independent predictors of severe WPL in our population were penetrating CD, anemia, diarrhea and a secondary level of education. The rate of WPL seems to be higher than the one reported in similar studies where it ranged from 25 to 42%, this may be due to design heterogeneity as some studies include admitted patients, others include only patients presenting to routine visits while others were based on online surveys [3,5,11]. Even though patients in remission had a significantly lower WPL rate than patients with active disease, WPL was still found in 21% of them with similar findings reported by Zand *et al.* and Cross *et al.* [6,19]. This can be due to irritable bowel syndrome (IBS) symptoms that patients might confuse with IBD symptoms. In fact, a meta-analysis published in 2020 has estimated the prevalence of IBS in patients with IBD to be 33.6% for patients in clinical remission and 23.5% for those achieving both clinical and endoscopic remission [20]. The study has always shown that patients with IBS were more likely to experience anxiety, depression and somatization. In addition, an Italian cross sectional study evaluating psychological changes experienced by IBD patients in remission has shown that patients’ quality of life is most affected by the presence of GI symptoms, mainly abdominal pain and diarrhea. The effect of these symptoms was in fact also extended to both social dimensions and physical activities [21]. These results, as well as ours suggest that inducing remission in IBD patients does not necessarily guarantee patients a good quality of life. At least one in five of those patients present with IBS symptoms which might critically affect their quality of life and thereby work productivity. On the other hand, none of the evidence-based interventions for IBS such as neuromodulators, probiotics and fecal transfer were assessed in patients with IBD reporting IBS symptoms. On the other hand, we have found significantly lower rates of absenteeism, presenteeism and WPL in patients with endoscopic remission. This corroborates data from previous studies that clearly associate disease activity and WPL [6,7]. However, it is worth mentioning that several studies established rapid and sustained beneficial effects of anti-TNF therapy on work productivity [22 23,24]. Regarding the low rate of remission in our study compared with the current literature, we believe that this can be, at least partially, due to the fact that we have chosen to consider endoscopic criteria when defining remission while in other similar studies only the patient reported outcomes were taken into consideration [6,25].

When exploring factors associated with severe WPL, diarrhea had the highest odds ratio (OR: 11.233, CI: 95% [3.630–34.762]). Similar results were reported by a French study targeting IBD patients working or with previous professional activities where the most disabling symptoms were diarrhea and fatigue. Diarrhea is in fact a very problematic symptom as not only it affects working pace but it also makes it challenging for patients to get to work place. Furthermore, anemia was identified in our study as an independent predictor of WPL. This result was expected as it has already been proven that anemia in IBD patients substantially compromise their of quality of life and their ability to focus and work [26]. We also established an association between secondary education level and severe WPL. We think that this might be linked to the nature of labor performed by this category. In fact, 53.4% of patients with a secondary level of education in our population are blue collar workers and 22.3% of them are day laborers. Both jobs are categorized by inflexible schedules, constraining conditions, high physical requirements and stressful work environments. Thus, IBD patients with these jobs might be more likely to miss work when not feeling well seen its high demands. When comparing the WPAI score features between CD and UC patients, we found that CD patients had significantly higher severe impact on daily life rates. This result is in accordance with similar studies; a meta-analysis published in 2018 found that quality of life in CD patients was significantly lower than in UC patients [1]. On the other hand, we found that patients with UC had a significantly higher severe absenteeism rates (13.1 vs 2%, p: 0.0017). We think that this might be explained by the disabling and restraining symptoms of UC such as diarrhea and bowel incontinence. In fact, causes for absenteeism such as feelings of shame and fear of frequent toilet visits were significantly more prevalent among UC patients. Moreover, this study highlighted the disparity between the substantial effect of IBD on patients’ productivity and the low rate of adjustments made by patients. In fact, only 7.9% of our population sought work relocations and only 11.6% changed their careers. Thus, it seems that IBD patients are either unaware of work adjustments possibilities or incapable of making them.

### Limitations & strengths

Some limitations of this study must be acknowledged. First, this is a monocentric study conducted in a tertiary hospital in Tunisia, this may lead to a selection bias as many of the patients referred to our center usually have a debilitating and refractory IBD. Moreover, it is important to acknowledge that the generalizability of our findings to the entire Tunisian population may be limited. While the study was conducted in a referral hospital that serves a significant portion of the central region of Tunisia, it is crucial to recognize that healthcare seeking behavior and patient characteristics and sociodemographic features can vary across different regions of the country. Therefore, caution should be exercised when attempting to apply our findings to the entire population of Tunisia. In addition, we did not have a control group to compare work productivity features to. Finally, our study did not explore the costs induced by WPL in IBD patients as this was beyond the scope of this study. On the other hand, this study focuses on the burden of IBD on work productivity in the Tunisian population, providing valuable insights into a population that has received limited attention in previous research. Tunisia, located in North Africa, possesses a unique demographic and cultural context that may influence the manifestation and impact of IBD. By investigating the work-related implications of IBD in this population, we can gain a better understanding of the challenges faced by Tunisian individuals living with IBD and tailor interventions and support systems accordingly. This study contributes to the growing body of literature on the global impact of IBD on work productivity while shedding light on the specific experiences of Tunisian patients, highlighting the importance of considering regional and cultural factors in managing the disease.

## Conclusion

In conclusion, this study highlights the substantial burden of IBD on work productivity among the Tunisian population. The high prevalence of WPL and frequent work absences demonstrate the significant impact of IBD symptoms, such as abdominal pain, diarrhea and rectal pain and bleeding, on the professional lives of patients. Moreover, the findings indicate that even when physically present at work, patients experience reduced productivity due to their IBD symptoms, as indicated by the high rate of presenteeism. We believe that gastroenterologists should be more aware of the impact of IBD on patients work life and that they should raise the issue with patients, guide them with proper individually tailored action plans and avoid unnecessary routine visits and procedures. In addition, studies evaluating irritable bowel syndrome treatments in IBD patients are warranted to improve quality of life of these patients.

**Summary points**

- Work productivity loss (WPL) was reported by 77.5% of the patients, indicating a high burden of inflammatory bowel disease (IBD) on their professional lives. This rate is higher than reported in similar studies, suggesting that the Tunisian population may experience a more significant impact on work productivity due to IBD.
- Among the patients, 47.8% had missed work in the past week due to IBD-related reasons, with a median absenteeism rate of 12.9%. This emphasizes the frequent need for work absences among IBD patients, which can be attributed to symptoms such as abdominal pain, diarrhea and rectal pain and bleeding.
- Presenteeism, the ability to be present at work while not fully productive, was reported by 71% of the patients, with a median presenteeism rate of 50%. This indicates that even when patients are physically present at work, their productivity is significantly impacted by their IBD symptoms.
- Severe absenteeism and WPL were reported by 5.1 and 39.1% of patients, respectively, indicating a subset of individuals experiencing substantial impairment in work productivity. Factors such as disease severity, anemia, and diarrhea were identified as independent predictors of severe WPL, emphasizing their significant impact on occupational functioning.
- Patients with UC had a significantly higher rate of severe absenteeism compared with CD patients (13.1 vs 2%). This difference may be attributed to the disabling symptoms associated with UC, such as bowel incontinence and frequent toilet visits, leading to increased work absences.
- Patients with a secondary level of education had higher rates of severe WPL, potentially due to the nature of labor performed by this group, including physically demanding and inflexible jobs. This suggests that the type of occupation may influence the impact of IBD on work productivity.
- Patients in clinical remission still reported a considerable rate of WPL (21%), which may be attributed to the presence of irritable bowel syndrome (IBS) like symptoms. IBS symptoms can coexist with IBD and significantly affect patients’ quality of life and work productivity, necessitating appropriate management strategies.
- The study highlights the disparity between the substantial impact of IBD on patients’ work productivity and the low rate of work adjustments made by patients. Only a small percentage of patients sought work relocations or changed their careers, indicating a potential lack of awareness or inability to make necessary accommodations.
- The findings underscore the need for gastroenterologists to be more aware of the impact of IBD on patients’ work lives, actively discuss these issues with patients, and provide individually tailored action plans. Additionally, further research evaluating treatments for IBS like symptoms in IBD patients is necessary to improve their quality of life and work productivity.
